# Multiparametric cardiovascular magnetic resonance assessment of cardiac allograft vasculopathy

**DOI:** 10.1186/1532-429X-16-S1-O3

**Published:** 2014-01-16

**Authors:** Christopher A Miller, Jaydeep Sarma, Josephine H Naish, Nizar Yonan, Simon G Williams, Steven M Shaw, David Clark, Keith Pearce, Martin Stout, Rahul Potluri, Alex Borg, Glyn Coutts, Saqib Chowdhary, Gerry P McCann, Geoffrey J Parker, Simon G Ray, Matthias Schmitt

**Affiliations:** 1 North West Heart Centre and The Transplant Centre, University Hospital of South Manchester, Manchester, UK; 2 Centre for Imaging Sciences & Biomedical Imaging Institute, University of Manchester, Manchester, UK; 3 Institute of Cardiovascular Sciences, University of Manchester, Manchester, UK; 4 Alliance Medical Cardiac MRI Unit, University Hospital of South Manchester, Manchester, UK; 5 Christie Medical Physics and Engineering, The Christie Hospital, Manchester, UK; 6 NIHR Leicester Cardiovascular Biomedical Research Unit and Department of Cardiovascular Sciences, University of Leicester, Leicester, UK

## Background

Cardiac allograft vasculopathy (CAV) continues to limit the long-term survival of heart transplant recipients. CAV affects both the epicardial arteries and the microvessels, however it does so independently, and epicardial and microvascular disease are both independently predictive of prognosis. Despite being associated with considerable limitations, coronary angiography has a class I recommendation for CAV surveillance and annual or biannual surveillance angiography is performed routinely in most centers. The aim of this study was to evaluate the diagnostic performance of multiparametric CMR in CAV, and to compare the performance of CMR to that of invasive coronary angiography, using contemporary invasive epicardial artery and microvascular assessment techniques as reference standards.

## Methods

All transplant recipients referred for surveillance angiography at a single UK transplant center over a 2-year period were prospectively screened for study eligibility. Patients prospectively underwent coronary angiography followed by coronary intravascular ultrasound (IVUS; epicardial artery reference standard) and index of microcirculatory resistance (IMR; microvascular reference standard). Within one month patients underwent multiparametric CMR. CMR assessment included LV volumetrics, circumferential strain and strain rate, torsion (circumferential-longitudinal shear), pixel-wise absolute myocardial blood flow quantification using generalized Tikhonov deconvolution with a b-spline representation of the impulse response function, late gadolinium enhancement and T1 mapping/extracellular volume measurement. Angiographic and CMR data were compared with the invasive epicardial artery (IVUS intima-media ("plaque") volume index) and microvascular (IMR) reference standards. In addition, 10 age- and sex-matched healthy volunteers underwent CMR.

## Results

**Figure 1:**
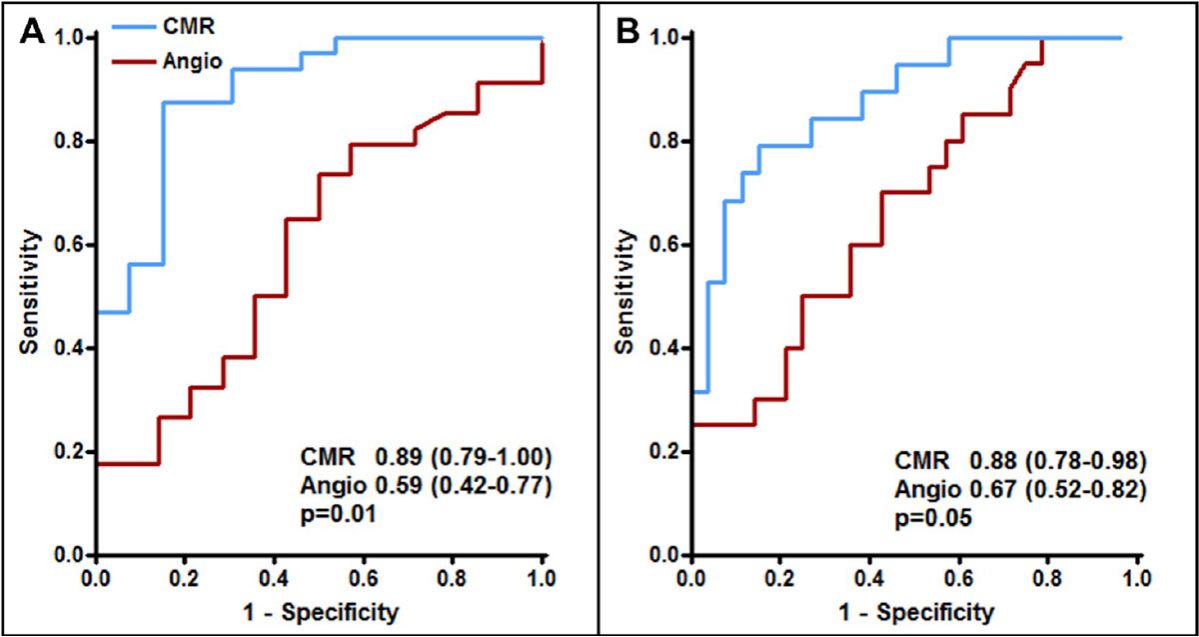
**Diagnostic performance of cardiovascular magnetic resonance myocardial perfusion reserve and angiography for detecting cardiac allograft vasculopathy (CAV)**. Diagnostic performance of cardiovascular magnetic resonance myocardial perfusion reserve (CMR) and angiography (angio) for detecting; (A) moderate cardiac allograft vasculopathy, defined as > median epicardial or microvascular disease; and (B) severe cardiac allograft vasculopathy, defined as > 75th centile epicardial or microvascular disease.

Forty-eight patients were recruited; median 7.1 years (IQR 4.6-10.3) since transplantation. Mean IVUS plaque volume index was 22.4 ± 9.8%; mean IMR was 23.7 ± 12.5. Selected univariable and multivariable associations between patient, angiographic and CMR data and IVUS plaque volume index and IMR are summarized in Table 1. CMR myocardial perfusion reserve was the only independent predictor of both epicardial (β = -0.57, p < 0.001) and microvascular disease (β = -0.60, p < 0.001) on stepwise multivariable regression. Myocardial perfusion reserve outperformed angiography for detecting moderate CAV (AUC 0.89, 95% confidence intervals 0.79-1.0 v 0.59 (0.42-0.77) respectively, p = 0.01; Figure 1A) and severe CAV (AUC 0.88 (0.78-0.98) v 0.67 (0.52-0.82), p = 0.05; Figure 1B).

**Table 1:** Associations between patient characteristics, angiography data and CMR data with invasive reference standards of epicardial artery disease (IVUS plaque volumes index; 1) and microvascular disease (IMR; 2).

1. Associations with coronary intravascular ultrasound (IVUS) plaque volume index			
Univariable associations	A. Patient characteristics	β	p value
	Time since transplantation	0.49	0.001
	Donor age	0.29	0.058
	B. Angiography data		
	Maximum angiographic stenosis	0.33	0.024
	C. CMR data		
	Early diastolic SR	-0.38	0.014
	MPR	-0.55	< 0.001
	Infarct LGE	0.35	0.022

Multivariable stepwise regression	A. Including patient characteristics and angiographic data		
	Time since transplantation	0.49	0.001
	B. Including patient characteristics and CMR data		
	Time since transplantation	0.47	< 0.001
	Early diastolic SR	-0.24	0.049
	MPR	-0.57	< 0.001

2. Associations with index of microcirculatory resistance (IMR)			

Univariable associations	A. Patient characteristics		
	Donor age	0.39	0.007
	Donor hypertension	0.35	0.016
	B. Angiography data		
	Maximum angiographic stenosis	-0.16	0.281
	C. CMR data		
	LVEF	-0.36	0.015
	εcc	0.46	0.002
	MPR	-0.55	< 0.001

Multivariable stepwise regression	Donor hypertension	0.29	0.012
	EF	-0.26	0.024
	MPR	-0.60	< 0.001

Only selected data is shown due to table size constraints. 1. On univariable analysis, maximum angiographic stenosis showed a significant association with plaque volume index, however after correcting for time since transplantation, this relationship was no longer significant. Early diastolic strain rate (SR), myocardial perfusion reserve (MPR) and infarct late gadolinium enhancement (LGE) were significantly associated with plaque volume index on univariable analyses, but only MPR and early diastolic SR remained independently associated with plaque volume index on multivariable analysis. 2. Maximum angiographic stenosis was not significantly associated with IMR on univariable analysis. Patient characteristics including donor age and donor hypertension showed significant associations with IMR on univariable analyses, as did CMR parameters such as LV ejection fraction (EF), peak systolic circumferential strain (εcc) and MPR. On multivariable analysis only donor hypertension, EF and MPR remained independently associated with IMR.

## Conclusions

CAV, including epicardial and microvascular components, can be detected more accurately using non-invasive CMR-based absolute myocardial blood flow assessment than with invasive coronary angiography, the current clinical surveillance technique.

## Funding

CAM is supported by a Fellowship from the National Institute for Health Research, UK (NIHR-DRF-2010-03-98). CAM, SGW, NY and MS have received research funding from New Start Transplant Charity, UK.

